# Local Treatment of Gleet by Compression

**Published:** 1860-09

**Authors:** G. P. Hachenberg

**Affiliations:** Springfield, Ohio


### ART. VI.—*On the Local Treatment of Gleet by Compression.* By G. P. HACHENBERG, M.D., of Springfield, Ohio.

IN my treatment of this often tedious malady, I have been induced to try the application of compression to relieve the inflamed or ulcerated urethra. This course being happily successful, I trust the publication of its results may prove of value, and be acceptable to the profession.

The obstinacy frequently met with in this disease may be due to the irritating character of the morbid secretion of the urethra, and the specific character of the inflammation, which, being of a low standard, deprives the affected parts of that recuperative power so manifest in most other parts of the body. I will not dwell upon the character of this inflammation; let it suffice that it exists, and that it demands a rather special treatment. How frequently do we witness the local application of absorbents, astringents, caustics, and other remedies, that for a time change the character of the inflammation, fail to remove the malady beyond its characteristic stage of torpidity! It is not difficult to perceive why many of the remedies administered to induce a new action of the affected parts may become the very agents of protracting rather than shortening the disease.

The more rational treatment of protracted cases of gleet in the male, is to combat the inflammation with our remedies, and carry it, as it were, from the stage we find it toward a cure, without exciting additional inflammation, under the idea of changing its character; a plan sometimes practicable in certain early stages, and in other parts of the body, but certainly not available in obstinate cases of gleet.

The remedy that will fulfill in a great measure the indication in question is gentle and prolonged compression by distention of the urethra. The instrument which I use for this purpose is made of different sizes, and is composed of ivory, or horn highly polished, and is simply a short bougie with a button or shoulder turned at one end to prevent it from slipping into the urethra.

The following method may be observed in its use: Before its introduction, at bedtime, the urethra should be well washed out with castile-soap and water, followed by a mildly astringent lotion. An instrument of a size, which will well fill the urethra, is then oiled, and with gentleness introduced. In a short time the passage will accurately and tenaciously grasp the instrument, and it is retained for the night without support or bandage. In the morning it is removed, followed by another cleansing process, which is repeated occasionally through the day. The application should be renewed every third or fourth night, until the cure is accomplished, which will occur after the third or sixth application. In removing the instrument in the morning there is sometimes a difficulty in getting it out of the urethra, so firmly is it held within its grasp. A gentle rotatory movement, however, will soon disengage it, its exit being then readily accomplished by traction.

The immediate effects of the use of this plan of treatment are uniformly of an efficient character. The first application often increases the tenderness of the parts, and even sometimes causes a slight hemorrhage; but a tolerance of the use of the instrument is soon established, until it no longer becomes a source of inconvenience. As this tolerance is established, we are favorably progressing with the cure of the case, for in the same ratio the discharge diminishes and soon ceases to show itself entirely.

In some cases, before the introduction of the instrument, I sprinkle it with calomel, tannin, or morphia, according to the indication. However, in the most of cases these topical applications are unnecessary.

The following cases are presented to illustrate more particularly the efficacy of this plan of treating gleet and certain forms of spermatorrhœa:—

CASE I.—In 1857, Mr. ——, aged twenty-five, of sedentary and temperate habits, had gonorrhœa for the second time, and, owing to neglect in the treatment, the disease terminated in obstinate gleet, which resisted many of the usual remedies for more than six months. Injections and blistering failed to give relief, and a cauterization was once resorted to, which greatly aggravated the disease. The bougie was likewise tried, medicated and otherwise, but without apparent benefit. A respite of all treatment was allowed for several days, when the compress was applied as described. The patient slept well through the night, and on removing the instrument in the morning there was some discharge of blood, accompanied with tenderness. In three or four days this had subsided, and the instrument used as before. Less tenderness and no bleeding followed this application. Three or four more applications were made at proper intervals, under which the discharge finally ceased, leaving the patient free from the disease ever since.

CASE II.—In the same year, Mr. ——, aged thirty-five, had gleet for nearly a year, for which he said he had been a patient at a Northern hospital for several months. The disease resulted from repeated attacks of gonorrhœa and a dissipated course of living. Temperance was enjoined, with physical and mental quietude; his bowels regulated with aperients, and the compression resorted to every fourth and fifth night for about three weeks. The discharge being purulent and viscid, the instrument was medicated with calomel with much benefit. The discharge gradually ceased, and the patient was dismissed. In this case there was a ready tolerance in the use of the instrument.

CASE III.—In 1858, Mr. —— was attacked with syphilis shortly before leaving California, and had nothing done for his relief until his arrival at this place. Under the usual local and constitutional treatment, the patient rapidly improved. After the healing of the external chancres, which were situated principally under the prepuce and on the gland, a gleety discharge followed, which proved to be of a very intractable character. Injections, cauterization with Lallemand's instrument, the bougie, and other remedies alike proved inefficient. Compression as described was finally resorted to every third night for two weeks, under which the local symptoms subsided, leaving the patient free from what we supposed to be a chancre within the urethra.

CASE IV.—A student was a victim to spermatorrhœa for many months, the result of the pernicious practice of onanism. Believing the nocturnal emission to be induced by the morbid sensibility, as well as the congested condition of the caput gallinaginis, I ordered compression to be applied to it by the common flexible bougie of large size. The instrument was well oiled, and carefully introduced into the urethra and retained for the night. This was repeated once a week for two months, to the complete relief of the patient. The use of the bougie caused little or no inconvenience.

